# Incremental value of left atrial booster and reservoir strain in predicting atrial fibrillation in patients with hypertrophic cardiomyopathy: a cardiovascular magnetic resonance study

**DOI:** 10.1186/s12968-021-00793-6

**Published:** 2021-10-11

**Authors:** Betty Raman, Robert W. Smillie, Masliza Mahmod, Kenneth Chan, Rina Ariga, Chrysovalantou Nikolaidou, Elizabeth Ormondroyd, Kate Thomson, Andrew R. Harper, Gifford Tan, Adam J. Lewandowski, Fernando Rodriguez Bajo, Eleanor C. Wicks, Barbara Casadei, Hugh Watkins, Stefan Neubauer

**Affiliations:** 1 University of Oxford Centre for Clinical Magnetic Resonance Research (OCMR), Division of Cardiovascular Medicine, Radcliffe Department of Medicine, University of Oxford, Oxfordshire, OX3 9DU United Kingdom; 2 Division of Cardiovascular Medicine, NIHR Oxford Biomedical Research Centre, University of Oxford, Oxford, UK

**Keywords:** Hypertrophic cardiomyopathy, Atrial fibrillation, Cardiovascular magnetic resonance imaging, Left atrial strain, Booster strain, Reservoir strain

## Abstract

**Background:**

Left atrial (LA) size and function are known predictors of new onset atrial fibrillation (AF) in hypertrophic cardiomyopathy (HCM) patients. Components of LA deformation including reservoir, conduit, and booster function provide additional information on atrial mechanics. Whether or not LA deformation can augment our ability to predict the risk of new onset AF in HCM patients beyond standard measurements is unknown.

**Methods:**

We assessed LA size, function, and deformation on cardiovascular magnetic resonance (CMR) in 238 genotyped HCM patients and compared this with twenty age, sex, blood pressure and body mass index matched control subjects. We further evaluated the determinants of new onset AF in HCM patients.

**Results:**

Compared to control subjects, HCM patients had higher LA antero-posterior diameter, lower LA ejection fraction and lower LA reservoir (19.9 [17.1, 22.2], 21.6 [19.9, 22.9], *P* = 0.047) and conduit strain (10.6 ± 4.4, 13.7 ± 3.3, *P* = 0.002). LA booster strain did not differ between healthy controls and HCM patients, but HCM patients who developed new onset AF (n = 33) had lower booster strain (7.6 ± 3.3, 9.5 ± 3.0, *P* = 0.001) than those that did not (n = 205). In separate multivariate models, age, LA ejection fraction, and LA booster and reservoir strain were each independent determinants of AF. Age ≥ 55 years was the strongest determinant (HR 6.62, 95% CI 2.79–15.70), followed by LA booster strain ≤ 8% (HR 3.69, 95% CI 1.81–7.52) and LA reservoir strain ≤ 18% (HR 2.56, 95% CI 1.24–5.27). Conventional markers of HCM phenotypic severity, age and sudden death risk factors were associated with LA strain components.

**Conclusions:**

LA strain components are impaired in HCM and, together with age, independently predicted the risk of new onset AF. Increasing age and phenotypic severity were associated with LA strain abnormalities. Our findings suggest that the routine assessment of LA strain components and consideration of age could augment LA size in predicting risk of AF, and potentially guide prophylactic anticoagulation use in HCM.

**Supplementary Information:**

The online version contains supplementary material available at 10.1186/s12968-021-00793-6.

## Background

At least 1 in 5 patients with hypertrophic cardiomyopathy (HCM) are affected by atrial fibrillation (AF) [1], the development of which heralds an unfavourable prognosis and is associated with a significantly higher all-cause mortality due to an increased risk of heart failure and stroke [1]. Current guidelines for HCM recommend a 48-h ambulatory Holter monitor every 2 years [2], though this may be inadequate for detecting new onset AF [3]. Lifelong anti-coagulation is recommended once AF is detected as cardioembolic risk is especially high in HCM. However, given the limitations of intermittent monitoring, anticoagulation sometimes follows an embolic complication. Therefore, the initiation of anti-coagulation therapy is clearly desirable when AF is anticipated.

Previous work has identified predictors for new onset AF in HCM. These include age [4], left atrial (LA) diameter [5], indexed LA end-diastolic volume (LAEDV) [6], and more recently, LA function [7]. However, contemporary guidelines have been slow to incorporate these biomarkers into the routine clinical management of HCM patients. Currently, the American Heart Association (AHA) recommends Holter monitoring for AF if a patient complains of palpitations [8]. The European Society of Cardiology (ESC) recommends intensification of arrhythmia surveillance when the anterior–posterior diameter of the LA exceeds 45 mm on echocardiography [2]. Specific recommendations for prophylactic anticoagulation are lacking and clinicians may initiate therapy once atrial diameter exceeds a given size in anticipation of AF. However, studies are emerging that show previously reported thresholds for LA size may be too conservative; with 59% of AF cases in one study occurring in patients with LA diameter < 45 mm [7]. More refined markers that determine AF risk are therefore clearly required.

The role of LA volume and diameter in predicting AF onset in HCM is well established. However, studies assessing the link between LA function and AF risk are limited. Determinants of LA function in HCM are complex and may include direct, myopathic aspects (where the mutant sarcomeric protein is expressed in atrial muscle) as well as secondary hemodynamic forces relating to increased atrial pressures in outflow obstruction, mitral regurgitation and diastolic dysfunction [9, 10]. LA function can be assessed using LA ejection fraction (LAEF) and/or LA deformation analysis on 2-dimensional (2D) cardiovascular magnetic resonance (CMR) feature tracking such as LA strain. LA strain consists of reservoir, conduit, and booster components. Whilst LA conduit strain is derived from the motion of atrial tissue during passive ventricular filling, LA reservoir strain and booster strain reflect passive and active LA functions, respectively [11, 12]. Comprehensive studies in HCM patients examining the association between AF and LA reservoir, conduit, and booster strain parameters on (CMR are lacking, yet LA strain components are emerging as sensitive markers for detecting impairment in LA function [13–17].

In this work, we set out to test the hypothesis that LA reservoir and booster strain on CMR will improve our ability to predict risk of incident AF. We further examined the determinants of LA strain components in HCM patients.

## Methods

### Study population and protocol

This is a retrospective analysis of data from an observational study approved by a local ethics committee (Reference: 07/Q1607/66, 12/LO/ 1979). Patients were recruited from the Inherited Cardiac Conditions (ICC) study at the University of Oxford between 2003 and 2016. As per the ESC diagnostic criteria, the recruitment for this study captured patients with genetically diagnosed or familial HCM who had wall thickness ≥ 13 mm [2], or non-familial HCM patients with wall thickness ≥ 15 mm but no other cause of hypertrophy identified. Genetic diagnosis used a minimum of 13-gene testing and patients were stratified based on genotype status (see Additional file 1 for details). Baseline characteristics including symptoms, comorbidities, and medications were retrieved from clinic letters. Patients with any history of AF at the time of CMR were excluded from this study. This exclusion was implemented after reviewing clinic letters and the results of a 24–48 h Holter carried out on each patient. Patients taking amiodarone were excluded. Patients with coronary artery disease, moderate to severe aortic stenosis, HCM phenocopies (amyloidosis, Fabry diseases, Danon disease), or contraindications to CMR were also excluded.

Twenty age, sex, blood pressure and body mass index (BMI) matched healthy subjects from another ethically approved study [18] provided normal values in light of potential variability in published normal ranges arising from inter-vendor strain differences. These healthy controls were free of known cardiovascular disease or family history of cardiac disease and were screened for AF on a 12-lead electrocardiogram (ECG). LA data (size, function, and strain) from age, sex and BMI matched controls were also specifically included to define the extent of impairment in LA strain components between HCM and non-HCM subjects.

### Cardiovascular magnetic resonance

**Fig. 1:**
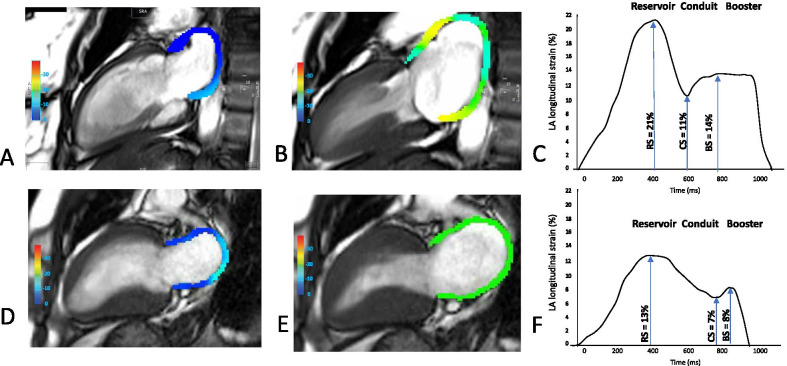
Left atrial longitudinal strain components in health and hypertrophic cardiomyopathy. Illustrative example of left atrial (LA) strain assessed in the vertical long axis view at the end of diastole using cardiovascular magnetic resonance (CMR) tissue tracking analysis from a healthy control subject and hypertrophic cardiomyopathy (HCM) (**A**, **D**). Peak LA longitudinal strain in the same control subject and HCM, respectively (**B**, **E**). Graph demonstrating the three functional elements of LA deformation in a healthy control subject (**C**). Graph demonstrating the three functional elements of LA deformation in a HCM patient (**F**)

CMR image acquisition including cine and late gadolinium enhancement (LGE) used established methods (see Additional file 1 for details). cvi42 software (Circle Cardiovascular Imaging Inc, Calgary, Alberta, Canada) was used as per established methods to assess left ventricular (LV) volume, function, mass and wall thickness and LA anterior–posterior diameter in the LV outflow tract (LVOT) view [19]. As previously described, LAEDV index, indexed LA end-systolic volume (LAESV), and LAEF were assessed from the horizontal and vertical long axis views [20, 21]. Similarly, LA peak longitudinal reservoir, conduit and booster strain were assessed using an automated tracking algorithm [22] (see Additional file 1 for details). 2-D feature-tracking strain analysis was averaged across the horizontal and vertical long axis views to derive strain components from cine images. Figure 1 depicts the aspect of this derivation from the vertical long axis. The LA image analysis was performed by an observer (RS) blinded to the clinical information or outcomes. LGE analysis was undertaken by setting the signal intensity threshold at six standard deviations above the mean intensity of a reference region of myocardium that had no visual evidence of enhancement [20].

### Clinical follow up

Patients in the Inherited Cardiac Conditions study, from which this study recruited, were followed up on a yearly basis either in clinic, or with a phone interview, and review of medical records and clinical letters if not followed-up in Oxford. The median follow-up time of those in this study was of 4.5 years. Follow-up was in line with clinical practice and consisted of a clinical visit and assessment, a 12-lead ECG, and 24–48 h Holter (2-yearly). The primary endpoint for this study was new onset AF, defined as an irregularly irregular heart rhythm with absent P-waves documented on a 12-lead ECG or 24–48 h Holter.

### Statistical analysis

Statistical analysis utilised R (version 3.5.0, R Foundation for Statistical Computing, Vienna, Austria). Packages used were: Survival, pROC, and time ROC. Normality of data was assessed by the Shapiro–Wilk and Anderson–Darling tests, visual inspection of quantile–quantile plots, and consideration for skew and kurtosis values. Mean (standard deviation/SD) and Median [Interquartile range/IQR] are presented accordingly for normally- and non-normally distributed data, respectively. Independent *t*-tests and Mann–Whitney tests were used for normally- and non-normally distributed data, respectively. Categorical variables are summarised as proportions (%) and the χ^2^ (with continuity correction) or Fisher’s exact tests were used to compare proportions. Inter- and intra-observer variability of strain measurements was determined by the interclass correlation coefficient, and coefficient of variation.

Stratification of the main LA parameters was based on the optimal thresholds (cut-off values) of these variables for predicting new onset AF, as calculated using Youden's index in conjunction with standard Receiver Operator Characteristic (ROC) analysis. For clinical applicability we rounded these thresholds.

Time-dependent ROC estimations were used to calculate C-statistics (concordance statistics), equivalent to the area under a ROC curve (AUC) and representative of the accuracy of a single value in the diagnosis of new onset AF at 3 years.

Univariate Cox proportional hazard regression analysis was performed to identify predictors of new onset AF. Relative risks were presented as hazard ratios (HR) with 95% confidence intervals (CI). Where appropriate, the proportional hazards assumption, presence of outliers, and linearity were tested for. Parameters under a significance threshold of *P* < 0.05 were included in the Cox multiple regression analysis. Kaplan–Meier cumulative survival curves free of AF were constructed for LA reservoir and booster strain values stratified by the rounded cut-offs. The survival curves were compared with the log-rank test. Simple linear and multiple regression was used to determine the variables that associate with LA strain. Unless adjustments were made for multiple tests, a p-value of *P* < 0.05 was considered significant throughout, and all tests were 2-sided.

The Bonferroni correction was applied selectively to the analysis in Table 1 and to the parameters included in the univariate Cox proportional hazard regression analysis to adjust ‘family-wise’ error rates in multiple comparisons of *related* groups and reduce the risk of type I error, whilst acknowledging the conservative nature of the correction. Correction was applied to the parameters relating to the LA (10 parameters in Table 1, giving an adjusted *P* of 0.05/10 = *P* < 0.005, and 15 parameters in the univariate analysis, giving an adjusted *P* of 0.05/15 = *P* < 0.0034), and separately to those relating to the LV (7 parameters in both analyses, giving an adjusted *P* of *P* < 0.0071). In the univariate analysis correction was also applied to the remaining baseline, clinical and medication parameters (28 parameters giving an adjusted *P* of *P* < 0.0018). By contrast the Bonferroni correction was not applied to the simple linear regression for the determinants of LA strain. This decision was made as this aspect of the study involved post-hoc testing of unplanned comparisons that we deem as hypotheses for further exploratory investigation and so avoiding a type II error was of greater importance [23].

**Table 1:** Baseline and CMR data: ‘controls vs HCM’; ‘No new onset AF vs new onset AF’

	Control subjects (n = 20)	HCM population, n = 238	*P* value	New onset AF (n = 33)	No new onset AF (n = 205)	*P* value
*Baseline and clinical*						
Age at CMR scan	48 [37, 64]	54 [43, 63]	0.362	61 [55, 66]	53 [41, 61]	0.001*
Men, n, %	15, 75	184, 77	1	25, 76	159, 78	0.824
Body mass index	26.6 [23.4, 27.8]	27.5 [24.6, 30.3]	0.145	28.5 [25.7, 30.8]	27.2 [24.5, 30.7]	0.129
Body surface area	1.9 (0.2)	2.0 (0.3)	0.276	2.1 [1.8, 2.2]	2.0 [1.8, 2.2]	0.51
Systolic blood pressure (mmHg)	128 (13.3)	131 (18.5)	0.558	130 (14.8)	131 (19.2)	0.968
Diastolic blood pressure (mmHg)	72 (8.0)	77 (10.3)	0.077	78 (9.7)	77 (10.4)	0.586
Hypertension, n, %	4, 20	99, 42	0.479	18, 55	81, 40	0.130
Mitral regurgitation class, %: no mitral regurgitation, 1, 2, 3, 4	0, 0, 0, 0, 0	71, 17, 7, 6, 0	–	73, 3, 15, 9, 0	71, 19, 5, 5, 0	0.014*
SCD score: ESC guidelines	N/A	2.0 (1.6)	–	2.3 (1.8)	2.0 (1.4)	0.273
VT historical, n, %	N/A	12, 5	–	5, 15	7, 3	0.015*
VT new onset, n, %	N/A	26, 11	–	6, 18	20, 10	0.225
TIA historical, n, %	N/A	6, 3	–	1, 3	5, 2	0.596
TIA new onset, n, %	0, 0	5, 2	–	2, 6	3, 1	0.143
Diabetes mellitus, n, %	0, 0	18, 8	–	2, 6	16, 8	1
Smoking history, n, %	-	59, 25	–	10, 30	49, 24	0.516
LGE presence, n, %	0, 0	185, 77	–	30, 91	155, 76	0.056
LGE mass (g)	0, 0%	15.0 [8.0, 26.0]	–	14.0 [6.9, 29.0]	16.0 [8.0, 26.0]	0.687
LGE (%)	0, 0%	10.5 [5.6, 15.5]	–	10.0 [4.8, 14.6]	10.7 [5.7, 15.5]	0.601
Latest NYHA class, %: 1, 2, 3, 4	20, 0, 0, 0	65, 28, 7, 0	–	44, 50, 6, 0	69, 25, 7, 0	0.012*
Sarcomeric variant present, n, %	N/A	78, 33	–	8, 24	70, 34	0.239
*Medications*						
Beta-blockers, n, %	0, 0	107, 45	< 0.001*	19, 58	88, 43	0.190
Calcium channel blockers, n, %	0, 0	59, 25	0.021	12, 36	47, 23	0.136
ACE-I/ARB, n, %	2, 10	57, 24	0.213	11, 33	46, 22	0.194
Diuretics, n, %	0, 0	9, 4	0.754	1, 3	8, 4	1
Aspirin, n, %	1, 5	81, 34	0.009*	11, 33	70, 34	0.845
Warfarin or other anticoagulation, n, %	0, 0	21, 9	0.315	11, 33	10, 5	< 0.001*
*CMR: left atrium*						
LA diameter (mm)	34.5 (4.3)	37.2 (5.7)	0.037	39.0 [34.0, 42.0]	37.0 [33.0, 40.0]	0.136
LAEDV index (mL/m^2^)	38.1 [35.9, 46.9]	43.8 [36.2, 51.1]	0.158	45.6 [39.4, 65.5]	43.5 [35.2, 50.7]	0.044
LAEDV (mL)	78.7 (20.4)	90.8 (31.7)	0.095	95.3 [77.7, 127.9]	84.5 [66.2, 105.7]	0.040
LAESV index (mL/m^2^)	17.3 (4.7)	23.6 (12.4)	0.025	31.5 (18.3)	22.3 (10.7)	< 0.001*
LAESV (mL)	33.5 (10.3)	46.9 (24.8)	0.017	62.9 (36.5)	44.28 (21.3)	< 0.001*
LA stroke volume (mL)	45.2 (12.9)	44.0 (15.2)	0.720	40.0 (16.9)	44.6 (14.9)	0.111
LA reservoir strain (%)	21.6 [19.9, 22.9]	19.9 [17.1, 22.2]	0.047	15.6 (5.4)	19.8 (4.3)	< 0.001*
LA conduit strain (%)	13.7 (3.3)	10.6 (4.4)	0.002*	8.6 (3.8)	10.9 (4.4)	0.004*
LA booster strain (%)	8.7 (1.8)	9.2 (3.1)	0.439	7.6 (3.3)	9.5 (3.0)	0.001*
LAEF (%)	57.2 [54.6, 61.5]	51.7 [44.8, 57.5]	0.001*	44.0 [26.7, 54.0]	52.5 [46.9, 58.0]	0.001*
*CMR: left ventricle*						
LV maximal wall thickness (mm)	–	19.0 [16.0, 22.0]	–	21.0 [18.0, 23.0]	19.0 [16.0, 22.0]	0.068
LVOT max pressure gradient (mmHg)	–	5.2 [3.8, 7.6]	–	5.4 [4.0, 8.1]	5.2 [3.8, 7.4]	0.698
LVOT obstruction ≥ 30 mmHg, n, %	–	9, 4	–	0, 0	9, 4	–
LVEDV index (mL/m^2^)	80.8 [65.9, 93.5]	71.1 [62.0, 82.1]	0.025	66.0 [55.0, 75.0]	72.2 [63.5, 82.3]	0.024
LVESV index (mL/m^2^)	29.0 [21.4, 35.0]	21.3 [17.0, 27.0]	0.008	19.3 [15.8, 25.0]	21.7 [17.6, 27.0]	0.039
LV stroke volume index (mL/m^2^)	51.4 [47.4, 60.0]	49.8 [43.8, 54.9]	0.171	45.4 [39.0, 53.2]	50.1 [44.0, 55.4]	0.066
LV ejection fraction (%)	64.9 [59.5, 71.2]	69.6 [65.0, 74.3]	0.024	70.6 (7.2)	69.3 (6.9)	0.292
LV mass (grams)	107.4 [88.8, 122.9]	151.5 [127.3, 183.5]	< 0.001*	178.8 [139.7, 200.4]	150.3 [123.7, 176.7]	0.024

Data are represented as mean ± standard deviation, or median [IQR]

Hypothesis testing via Student’s *t*-test or Mann–Whitney U test, as appropriate

*ACEI* angiotensin converting enzyme inhibitor, *ARB* angiotensin receptor blocker, *ESC* European Society of Cardiology, *LA* left atria, *LAEDV* left atrial end diastolic volume, *LAESV* left atrial end systolic volume, *LGE* late gadolinium enhancement (5-SD), *LGE %* percentage of tissue enhanced by gadolinium, *LV* left ventricle, *LVEDV* left ventricular end diastolic volume, *LVESV* left ventricular end systolic volume, *LVOT* left ventricular outflow tract, LVOT obstruction, when pressure ≥ 30 mmHg, *NYHA* New York Heart Association, *SCD* sudden cardiac death, *TIA* transient ischemic attack, *VT* ventricular tachycardia

*Indicates a significant difference. This significance level is *P* < 0.05 without Bonferroni correction for baseline clinical variables and medications, whereas with Bonferroni correction the significance values are *P* < 0.005 and *P* < 0.0071 for CMR: Left Atrium and CMR: Left Ventricle, respectively

## Results

### Reproducibility of LA strain measurements

Inter-observer (GT) and intra-observer variability of LA strain were within the clinically acceptable range (refer to Additional file 1 and Table S1 for details).

### Baseline characteristics of HCM patients and controls

Of the 281 patients screened for study inclusion, 24 (9%) were excluded due to pre-existing AF at the time of the CMR scan, 14 (5%) were excluded due to taking amiodarone, and 5 (2%) were excluded due to issues with data collection, leaving 238 HCM patients in the study (see Additional file 1: Fig. S1).

The median subject age was 54 years with IQR[43, 63]), 184 (77%) were men, 107 (47%) were on beta blockers, the average 5-year risk of sudden cardiac death (SCD) was low based on the ESC guidelines for risk stratification (2.0 ± 1.6%), 69 (29%) had mitral regurgitation (81% of which was mild or moderate), and 9 (4%) had LVOT obstruction ≥ 30 mmHg. Thirty-three (14%) developed new onset AF during follow-up after a mean time of 3.6 years (median of  3.0 years with IQR [2.8, 6.8]). Of these, 11 were in persistent AF whereas 22 had evidence of paroxysmal AF. The baseline clinical characteristics of patients and healthy controls are summarised in Table 1.

As expected, when compared to controls, HCM patients had smaller indexed LV end-diastolic and end-systolic volumes, and higher LV ejection fraction (LVEF) and LV mass (Table 1), although after application of the Bonferroni correction only LV mass differed significantly. LA diameter in the LVOT view and LAESV index were higher in HCM than controls, whilst LAEF, reservoir and conduit strain were lower in HCM (Fig. 1) although of these findings, only those relating to conduit strain and LAEF were consistent after the Bonferroni correction was applied. There was a trend towards LAEDV index being increased in HCM, whereas consistent with a previous study, LA booster strain did not differ between HCM and healthy controls even at a significant level of *P* < 0.05 [17].

### Patients with and without new onset AF

HCM patients who developed new onset AF were significantly older and were more likely to have mitral regurgitation, higher New York Heart Association (NYHA) scores, and non-sustained ventricular tachycardia (VT). Patients with new onset AF had higher LV wall thickness, LV mass, LAEDV index, LAESV index, and more impaired LAEF and global LA strain (reservoir, conduit, and booster) at baseline CMR than those who did not (at a *P* < 0.05 level). There was a non-significant trend towards a higher LA anterior–posterior diameter at baseline in patients who developed AF versus those who did not (*P* = 0.136). Once the Bonferroni correction was applied (Table 1), only LAESV index, LAEF and global LA strain parameters remained significantly different between groups.

### Diagnostic accuracy of LA parameters in predicting new onset AF in HCM

The optimal threshold values for predicting new onset AF for the main LA parameters and age were calculated using ROC curve analysis. Additional file 1: Table S2 shows rounded optimal thresholds and measurements of diagnostic accuracy (C-statistic).

### Biomarkers associated with incident AF in HCM

To assess the determinants of AF in HCM patients, we undertook a univariate analysis (see Table 2) and found the following variables to be significant predictors of new onset AF once Bonferroni corrections were applied: age at scan, LAESV index, LA reservoir, conduit, and booster strain, and LAEF. Of note, LA diameter (antero-posterior) assessed to be ≥ 45 mm on CMR (a threshold derived from transthoracic echo studies) was not significantly associated with new onset AF in our cohort even at a conventional *P* value of 0.05 (*P* = 0.348).

**Table 2:** Univariate predictors of new onset AF in all patients

	Univariate analysis	
	HR (95% CI)	P value prior to correction
*Baseline and clinical*		
Age at scan, per year	1.07 (1.04–1.10)	< 0.001*
Age ≥ 55 years (rounded ROC threshold)	5.94 (2.50–14.09)	< 0.001*
Male sex	0.95 (0.43–2.11)	0.903
Systolic blood pressure, per mmHg	1.01 (0.99–1.03)	0.341
Diastolic blood pressure, per mmHg	1.03 (0.99–1.06)	0.155
Body mass index, per unit	1.03(0.96–1.12)	0.393
Hypertension	2.00 (0.99–4.03)	0.051
Diabetes	0.73(0.17–3.07)	0.669
Smoking history	1.16 (0.55–2.45)	0.692
Hypercholesterolemia	1.04 (0.36–2.96)	0.947
Family history of SCD 1st degree relative	0.45 (0.17–2.23)	0.454
Family history of SCD 2nd degree relative	1.21 (0.42–3.46)	0.725
SCD risk score (ESC)	1.04 (0.86–1.26)	0.673
VT	1.43 (0.59–3.46)	0.433
TIA stroke	2.92 (0.69–12.31)	0.145
Syncope	0.48 (0.11–2.02)	0.318
NYHA class	1.79 (1.09–2.93)	0.021
LGE, presence or absence	6.27 (0.85–46.05)	0.071
LGE mass, g	1.00 (0.98–1.02)	0.871
LGE, %	1.00 (0.96–1.04)	0.926
Presence of sarcomeric variant	0.52 (0.23–1.17)	0.113
Mitral regurgitation presence (any grade) vs absence	1.16 (0.81–1.67)	0.407
Mitral regurgitation grade 1/nomitral regurgitation vs grade 2/3/4	2.16 (0.97–4.79)	0.058
*Medication*		
Beta-blockers	1.22 (0.61–2.44)	0.577
Calcium channel blockers	1.96 (0.95–4.02)	0.067
ACE-I/ARB	1.61 (0.78–3.34)	0.201
Diuretics	0.88 (0.12–6.46)	0.899
Aspirin	1.00 (0.48–2.09)	0.994
*CMR: LA*		
LA diameter, mm, continuous	1.05 (0.99–1.12)	0.046
LA diameter ≥ 45 mm (ESC threshold)	1.58 (0.61–4.12)	0.348
LA diameter ≥ 42 mm (ROC threshold rounded)	2.11 (1.05–4.25)	0.036
LAEDV index, mL/m^2^, continuous	1.02 (1.01–1.04)	0.023
LAEDV index ≥ 50 mL/m^2^ (ROC threshold rounded)	2.15 (1.08–4.27)	0.029
LAESV index, mL/m^2^, continuous	1.04 (1.02–1.06)	< 0.001*
LAESV index ≥ 27 mL/m^2^ (ROC threshold rounded)	2.42 (1.22–4.82)	0.012
LA reservoir strain, % (continuous)	0.86 (0.81–0.91)	< 0.001*
LA reservoir strain ≤ 18% (ROC threshold rounded)	3.64 (1.81–7.32)	< 0.001*
LA conduit strain, %, continuous	0.88 (0.81–0.95)	0.002*
LA conduit strain ≤ 12% (ROC threshold rounded)	3.15 (1.29–7.72)	0.012
LA booster strain, %, continuous	0.87 (0.78–0.97)	0.014
LA booster strain ≤ 8% (ROC threshold rounded)	2.93 (1.44–5.95)	0.003*
LAEF (%) continuous	0.94 (0.92–0.97)	< 0.001*
LAEF ≤ 45%, (ROC threshold rounded)	3.42 (1.72–6.78)	< 0.001*
*CMR: left ventricle*		
LV maximal wall thickness, mm	1.06 (1.00–1.14)	0.059
LV mass, grams	1.00 (1.00–1.01)	0.180
LVOT max pressure gradient, mmHg	1.04 (0.95–1.14)	0.350
LVEDV index (mL/m^2^)	0.98 (0.95–1.00)	0.069
LVESV index (mL/m^2^)	0.96 (0.91–1.01)	0.114
LV stroke volume index, mL/m^2^	0.98 (0.94–1.01)	0.163
LV ejection fraction, %	1.02 (0.97–1.07)	0.506

*LA* left atria, *LAEDV* left atrial end diastolic volume, *LAEF* left atrial ejection fraction, *LAESV* left atrial end systolic volume, *LGE* late gadolinium enhancement (5-SD), *LGE %* percentage of tissue enhanced by gadolinium, *LV* left ventricle, *LVOT* left ventricular outflow tract, *LVEDV* left ventricular end diastolic volume, *LVESV* left ventricular end systolic volume, *LVSV* left ventricular stroke volume, *NYHA* New York Heart Association, *SCD* sudden cardiac death, *TIA* transient ischemic attack, *VT* ventricular tachycardia

*Indicates a significant difference given Bonferroni correction to the *P* value as stated in the methods

When undertaking Cox multiple regression analysis, we considered each of the five main LA parameters that displayed evidence of significance in the Bonferroni corrected Univariate analysis. For completeness, LA diameter was also analysed in the multiple regression analysis despite not reaching significance. A model was initially constructed for each main LA parameter to avoid collinearity between them (six models shown in Table 3). Age was included in all models due to reaching significance in univariate analysis. When analysing all HCM patients, age (≥ 55 years) was a significant predictor of new onset AF, with hazard ratios (HR) that ranged from 4.05 to 6.30. LA EF (≤ 45%) was associated with a twofold increased risk of new onset AF (Table 3). LA reservoir strain (≤ 18%) and booster strain (≤ 8%) were associated with a nearly threefold and fourfold increased risk of new onset AF. LAESV index, and conduit strain were not significant predictors.

**Table 3:** Six multiple regression models: determinants of AF in all HCM patients, and in those with LA diameter < 45mm, indicating a low risk for AF development

	Model 1	Model 2	Model 3	Model 4	Model 5	Model 6
	LA diameter	LAESV index	LA reservoir strain	LA conduit strain	LA booster strain	LAEF
	HR (95% CI)	HR (95% CI)	HR (95% CI)	HR (95% CI)	HR (95% CI)	HR (95% CI)
All HCM patients, n = 238						
Age ≥ 55	5.07 (2.23–11.55)**	4.72 (2.06–10.95)**	4.05 (1.73–9.46)**	4.42 (1.76–11.06)**	6.62 (2.79–15.70)**	4.19 (1.80–9.76)**
LA diameter ≥ 42 mm	1.92 (0.95–3.91)	–	–	–	–	–
LAESV index ≥ 27 mL/m^2^	–	1.82 (0.90–3.68)	–	–	–	–
LA reservoir strain ≤ 18%	–	–	2.56 (1.24–5.27)*	–	–	–
LA conduit strain ≤ 12%	–	–	–	1.58 (0.58–4.25)	–	–
LA booster strain ≤ 8%	–	–	–	–	3.69 (1.81–7.52)**	–
LAEF ≤ 45%	–	–	–	–	–	2.43 (1.20–4.92)*
HCM patients with LA diameter < 45 mm, n = 217						
Age ≥ 55	4.05 (1.72–9.51)**	4.73 (2.04–10.96)**	3.34 (1.38–8.06)**	3.41 (1.30–8.96)*	5.78 (2.35–14.23)**	3.67 (1.53–8.81)**
LA diameter ≥ 42 mm	1.94 (0.84–4.47)	–	–	–	–	–
LAESV index ≥ mL/m^2^	–	1.82 (0.90–3.68)	–	–	–	–
LA reservoir strain ≤ 18%	–	–	2.49 (1.13–5.32)*	–	–	–
LA conduit strain ≤ 12%	–	–	–	1.72 (0.60–4.90)	–	–
LA booster strain ≤ 8%	–	–	–	–	3.74 (1.70–8.07)**	–
LAEF ≤ 45%	–	–	–	–	–	1.98 (0.92–4.28)

*EF*, ejection fraction, *HCM*, hypertrophic cardiomyopathy, *HR*, hazard ratio, *LA*, left atria, *LAEDV*, left atrial end diastolic volume, *LAEF*, left atrial ejection fraction

*Indicates a significant difference of P < 0.05

**Indicates a significant difference of *P* < 0.01

Further to this, we applied the same six Cox multiple regression models to a subgroup of HCM patients in whom LA diameter was less than 45 mm (n = 217), a group that would otherwise not be considered at risk of AF based on ESC guidelines [2]. Age was again a significant predictor of AF in each model. Of the LA parameters, LA reservoir (HR 2.49, CI 1.13–5.32, *P* = 0.023) and booster (HR 3.74, CI 1.70–8.07, *P* ≤ 0.001) strain were the only variables to retain significance (Table 3).

We then included all the parameters that retained significance in the Cox multiple regression models on all HCM patients in a model (Table 4). In this, only age and LA booster function retained significance, with age being a more powerful predictor than booster strain (HR of 5.22 vs 3.08).

**Table 4:** Multiple regression: predicting new onset AF in all HCM patients

	HR (95% CI)	P value
Age ≥ 55 years	5.22 (2.12–12.83)	< 0.001**
LA reservoir strain ≤ 18%	1.21 (0.44–3.32)	0.717
LA booster strain ≤ 8%	3.08 (1.43–6.64)	0.004**
LAEF ≤ 45%	1.73 (0.68–4.40)	0.252

*LAEF* left atrial ejection fraction, *HR* hazard ratio, *LA* left atria, *LAEDV* left atrial end diastolic volume

*Indicates a significant difference of P < 0.05

**Indicates a significant difference of *P* < 0.01

### Survival analysis

**Fig. 2:**
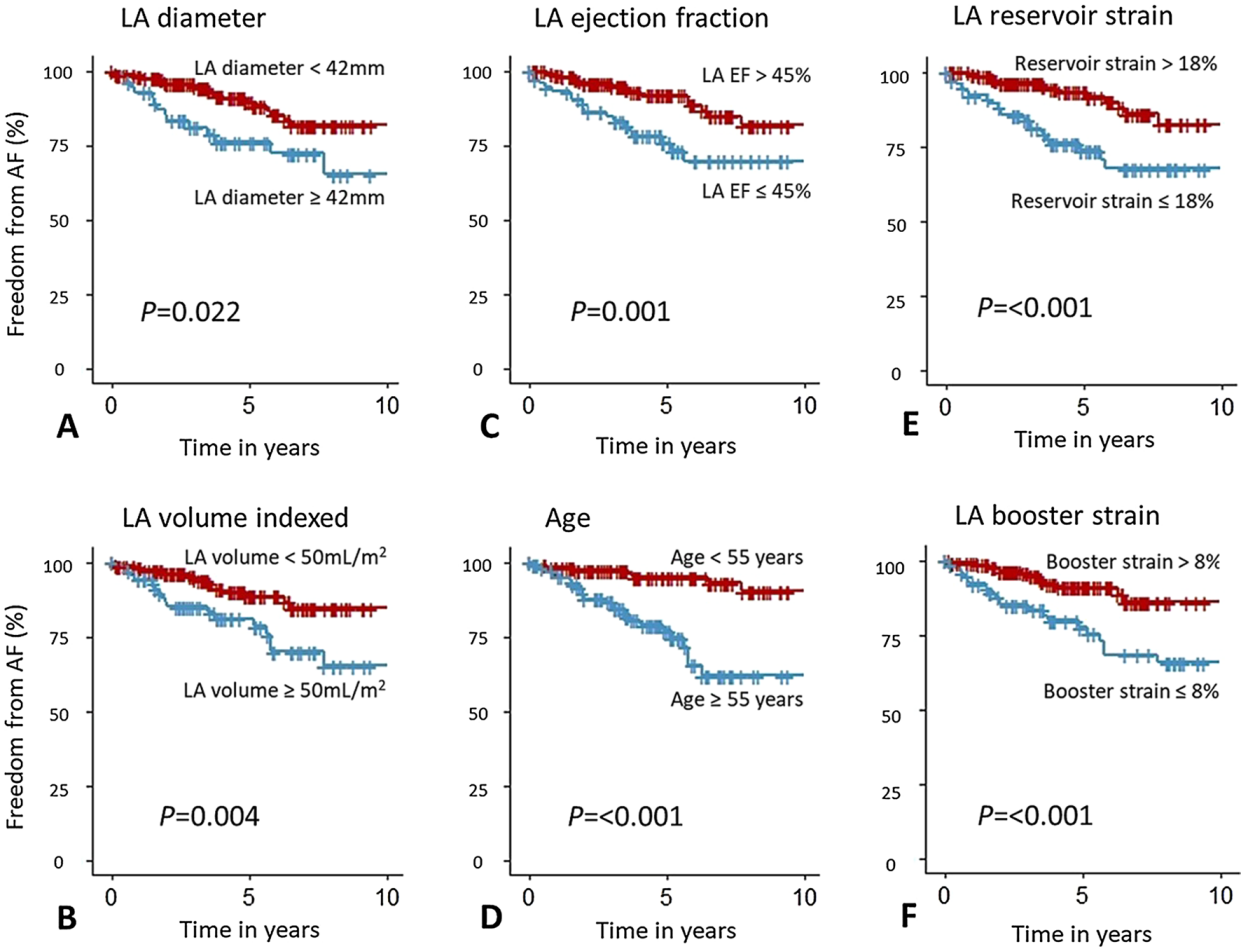
Kaplan Meier curves demonstrating freedom from atrial fibrillation for HCM patients. Survival curves of all HCM patients (n = 238) showing freedom from atrial fibrillation (AF) when stratified according to the variables included in the Cox multiple regression analysis: **A** Left atrial (LA) diameter; **B** LA end diastolic volume (LAEDV) index; **C** LA ejection fraction; **D** Age ; **E** LA reservoir strain; **F** LA booster strain

Survival graphs displaying survival free from AF were produced for the variables that reached significance in the multiple regression models (Fig. 2). There was a significant difference in freedom from AF in the 10 years post follow up when stratifying by LA diameter, LAEDV index, LAEF, and in particular age. When all the HCM cases were stratified by an LA reservoir strain of 18%, the 5 years survival free of AF was 76% for those with reservoir strain ≤ 18%, compared to 96% in those with strain > 18%. Similarly, when stratifying with LA booster strain of 8%, the 5 years survival free of AF was 81% in those with booster strain ≤ 8%, compared to 94% in those with booster strain > 8%.

### Determinants of LA strain components

Univariate and multivariable determinants of LA reservoir, conduit, and booster strain are shown in Table 5. On multivariable regression analysis, age at scan associated with all three of reservoir, conduit, and booster strain. In addition, NYHA class, LV maximal wall thickness, LV mass, and LV ejection fraction (LVEF) also remained significantly associated with reservoir strain. Presence of LGE, mitral regurgitation, LV mass, and LV stroke volume also independently associated with conduit strain, whilst a history of smoking also associated with booster strain.

**Table 5:** Univariate and multiple linear regression showing determinants of LA strain in an AF naïve population

	Univariate linear regression						Multivariable linear regression					
	Reservoir strain		Conduit strain		Booster strain		Reservoir strain		Conduit strain		Booster strain	
	β	P values	β	P values	β	P values	β	P values	β	P values	β	P values
Age, per year	− 0.11	< 0.001*	− 0.19	< 0.001*	0.06	< 0.001*	− 0.10	< 0.001*	− 0.18	< 0.001*	0.04	0.018*
Gender	1.17	0.102	1.45	0.050	0.66	0.196	–	–	–	–	–	–
Systolic blood pressure, per mmHg	− 0.06	0.001*	− 0.07	< 0.001*	0.01	0.567	0.01	0.637	− 0.01	0.891	–	–
Diastolic blood pressure, per mmHg	− 0.07	0.027*	− 0.07	0.052	− 0.01	0.599	− 0.04	0.309	–	–	–	–
Body mass index, per unit	− 0.10	0.120	− 0.16	0.024*	0.06	0.245	–	–	0.11	0.095	–	–
Hypertension	− 0.52	0.392	− 2.06	0.001*	1.29	0.003*	–	–	0.71	0.272	0.01	0.998
Diabetes	0.07	0.951	− 2.36	0.044*	2.00	0.014*	–	–	− 0.21	0.834	0.65	0.473
Smoking history	0.31	0.655	− 1.38	0.056	1.59	0.001*	–	–	–	–	1.33	0.014*
Hypercholesterolemia	− 0.46	0.626	− 2.20	0.024*	1.24	0.066	–	–	− 0.21	0.771	–	–
Familyhistory of SCD 1st degree relative	0.67	0.566	0.24	0.843	0.68	0.424	–	–	–	–	–	–
Family history of SCD 2nd degree relative	0.56	0.540	0.26	0.786	0.34	0.613	–	–	–	–	–	–
SCD risk score	− 0.24	0.602	− 0.85	0.067	0.56	0.080	–	–	–	–	–	–
VT	0.17	0.864	− 1.19	0.257	1.49	0.038*	–	–	–	–	1.24	0.093
TIA stroke	− 1.83	0.461	− 1.98	0.442	− 0.17	0.923	–	–	–	–	–	–
Syncope	− 1.40	0.088	− 2.34	0.006*	0.60	0.313	–	–	− 0.70	0.376	–	–
NYHA class	− 1.84	< 0.001*	− 2.63	< 0.001*	0.35	0.325	− 1.07	0.042*	− 0.52	0.274	–	–
LGE, presence/absence	− 2.15	0.012*	− 3.88	< 0.001*	1.33	0.030*	− 0.93	0.256	− 1.86	0.007*	0.71	0.254
LGE, %	0.02	0.561	− 0.05	0.123	0.05	0.028*	–	–	–	–	0.02	0.368
LGE mass, grams	− 0.03	0.166	− 0.05	0.017*	0.01	0.409	–	–	− 0.02	0.186	–	–
Presence of sarcomeric variant	0.88	0.166	2.49	< 0.001*	− 1.37	0.002*	–	–	0.24	0.722	− 0.72	0.215
Mitral regurgitation	− 3.26	0.001*	− 4.12	< 0.001*	0.16	0.816	− 1.16	0.256	− 2.89	0.001*	–	–
LV maximal wall thickness, mm	− 0.32	< 0.001*	− 0.32	< 0.001*	− 0.05	0.386	− 0.20	0.021*	− 0.15	0.052	–	–
LV mass, grams	− 0.03	< 0.001*	− 0.02	0.003*	− 0.01	0.068	− 0.01	0.035*	− 0.01	0.030*	–	–
LVOT max pressure gradient, mmHg	0.08	0.462	− 0.12	0.282	0.13	0.101	–	–	–	–	–	–
LVOT obstruction ≥ 30 mmHg, n, %	− 0.88	0.271	− 1.01	0.223	− 0.23	0.693	–	–	–	–	–	–
LVEDV index, mL/m^2^	0.02	0.383	0.06	0.003*	− 0.03	0.028*	–	–	− 0.04	0.278	− 0.02	0.370
LVESV index, mL/m^2^	− 0.03	0.385	0.03	0.452	− 0.05	0.088	–	–	–	–	–	–
LV stroke volume index, mL/m^2^	0.06	0.050	0.12	< 0.001*	− 0.04	0.055	–	–	0.13	0.011*	–	–
LV ejection Fraction, %	0.10	0.024*	0.08	0.076	0.02	0.589	0.131	0.002*	–	–	–	–

*LGE* late gadolinium enhancement (5-SD), *LV* left ventricle, *LVEDV* left ventricular end diastolic volume index, *LVESV* left ventricular end systolic volume index, *LVOT* left ventricular outflow tract, *LVSV* left ventricular stroke volume, *NYHA* New York heart association, *SCD* sudden cardiac death, *TIA* transient ischemic attack, *VT* ventricular tachycardia

*Indicates a significant difference of P < 0.05

## Discussion

In this study, we used CMR to assess the role of LA deformation when compared to standard LA parameters and baseline characteristics in predicting new onset AF in HCM. In addition to age and LAEF, we have shown that LA reservoir and booster strain have the ability to augment prediction of new onset AF in HCM. Specifically, we show that LA reservoir and booster strain dysfunction are important determinants of AF risk even in those with an LA diameter less than 45 mm, a threshold set by the ESC to escalate arrhythmia surveillance frequency. Of interest, a marginally higher risk of incident AF was specifically seen in those with booster strain dysfunction compared to other parameters. We also determined the factors that associate with markers of atrial deformation and show that age and phenotypic severity adversely influenced LA deformation.

The prediction of new onset AF in HCM allows for intensification of monitoring and prophylactic use of anticoagulation in those at high risk, thus minimising the effects of subsequent harmful sequelae, primarily stroke, myocardial infarction (embolic), and heart failure [24]. Early studies investigating the determinants of new onset AF have found LA size to be important in guiding surveillance. More recently, LA function has emerged as a useful determinant of new onset AF in HCM patients [4–7]. LA deformation or strain provides an additional measure of atrial mechanics and has been found to provide deeper insights into the risk of arrhythmia, embolic events, and other adverse outcomes in some cardiovascular diseases [25, 26]. In patients with AF, Hsu et al. demonstrated an independent association between LA strain and risk of embolic complications such as stroke [27].

Most studies to date have used transthoracic echocardiography (TTE) for the assessment of LA strain in HCM [14, 15]. However, TTE remains limited in its ability to provide consistently high quality images which are often confounded by body habitus, comorbidities such as chronic obstructive lung disease, and operator expertise [28]. In comparison, CMR provides an excellent platform to precisely and reproducibly evaluate cardiac chambers, providing additional information on myocardial tissue characteristics [29]. The high resolution images acquired by CMR enable retrospective analyses of LA dimensions and function from both LA and right atria, which are well visualised in standard cardiac planes acquired in accordance with consensus guidelines [30]. In spite of the increasing use of CMR for assessment of HCM patients, studies examining the additive value of LA strain assessment on CMR in AF prediction in HCM are lacking.

Here, we systematically examined components of LA strain as assessed on CMR, in addition to standard metrics of LA size and function. Consistent with previous studies, we found LA reservoir and conduit strain to be impaired in HCM patients [13, 31], though after correcting for multiple comparisons only conduit strain remained impaired. By contrast, LA booster strain, the active component of LA deformation, was not significantly different between HCM patients and healthy control subjects. Indeed, the evidence in support of booster dysfunction in HCM patients is conflicting. In a study by Yang and colleagues [17], LA booster strain was not different between non-obstructive HCM patients and controls. In contrast, Kowallick et al. reported a significant impairment in LA booster strain in HCM patients relative to controls [32].

A number of studies have postulated that booster function could indicate an increase in fibrotic burden of the atria [33, 34]. In a recent study by Sivalokanathan et al. [35], atrial fibrosis as detected by atrial LGE was found to be greater in those with new onset AF [35]. Likewise, in our study booster strain was reduced in those patients that developed AF compared to those that did not. Thus, the relationship between booster strain and AF may potentially reflect an increased burden of atrial fibrosis or an underlying atrial myopathy in HCM patients.

The absolute values of LA strain reported in this study were lower than those reported elsewhere [36], though comparable with some studies using CMR myocardial feature tracking. Values from healthy, yet elderly controls in studies by Evin et al. [37] and Lamy et al. [38] matched our relatively old and obese control group closely [37, 38]. The values from all HCM patients in this study are within reasonable range of those presented by Sivalokanathan et al. and Kowallick [13, 35]. In the present study, LA diameter [5], volume [6] and LAEF [7] were univariate determinants of AF risk, largely consistent with earlier work. We additionally showed that LA reservoir and booster strain are associated with a threefold and fourfold increase in new onset AF risk, respectively, and, as illustrated by the freedom-from-AF survival curves (see Fig. 2 and Additional file 1: Fig. S2), both measures discriminated risk of new onset AF with reasonable diagnostic accuracies (see Additional file 1: Table S2). Of importance, in patients with LA diameter of less than 45 mm, booster and reservoir strain remained strong independent determinants of new onset AF.

To date, there have been a number of studies of HCM patients in which age independently predicted AF risk [4, 24, 39]. Concordantly, we also found age to be a strong determinant of future risk of new onset AF. Consensus guidelines make no specific recommendations for prophylactic anticoagulation or frequency of arrhythmia monitoring in patients based on age. Our findings highlight the need to incorporate age into future risk prediction models being developed for AF or stroke risk prediction in patients with HCM and provide an argument to consider prophylactic anticoagulation therapy in elderly HCM patients who are not at risk of significant bleeding.

Wider evidence surrounding the impact of sarcomeric mutations on AF incidence is mixed; Bongini et al., found no association between HCM genetic subtype and AF [40], whereas Lee et al. report myosin heavy chain—7 (*MYH7)* to be predictive of AF [41]. In this study, the presence of sarcomeric variant trended towards associating with lower risk of AF on univariate analysis (HR of 0.52, *P* = 0.113). We believe that this may have been due to a higher prevalence of hypertension and increased age among the sarcomere negative HCM patients (Additional file 1: Table S3). Such differences in baseline characteristics of HCM patients with and without sarcomeric variants have also been observed in a recent multicentre CMR registry of HCM (HCMR study) [42].

This study assessed the determinants of LA strain in an AF naïve HCM population. Our findings in the multiple regression models suggest that age, markers of phenotypic severity (LV wall thickness, mass, mitral regurgitation, LGE) and baseline SCD risk are associated with adverse LA mechanical remodelling and could explain the emerging prognostic implications of LA strain for many cardiovascular events [11, 14, 26]. That age was negatively associated with reservoir and conduit strain and positively associated with booster strain has been noted previously in non-HCM populations—atrial emptying is more dependent on booster function in older age [43, 44]. The negative correlation between LGE and conduit strain and LV mass/wall thickness and reservoir strain in HCM is also not surprising given that both conduit and reservoir largely reflect LA compliance, which may be impacted by the burden of fibrosis and adverse remodelling [45]. Although the presence of sarcomeric variant was seen to associate with both conduit and booster strain on univariate analysis, this failed to reach statistical significance in our multivariable model. This may be because of the differences in baseline characteristics (in particular age and hypertension) of sarcomere positive and negative HCM patients. Further studies are needed to tease out the precise relationship between sarcomeric variant status and LA strain components.

## Limitations

This is a retrospective single-centre study, and thus some characteristics, such as the extent of LVOT obstruction, are not reflective of the population frequency. Temporal resolution of cine imaging was not consistent (39–60 ms) in all patients (due to the retrospective nature of this study) and could in theory contribute to variability in strain measurements. Prior to the publication of ESC guidelines in 2014, TTE analysis was only done as per clinical need. Therefore, we lacked the ability to compare or correlate CMR and TTE data. Similarly, diastolic function was not available on the same day as CMR for many patients. A dedicated atrial stack on LGE imaging was not undertaken and therefore fibrosis in the atria could not be assessed. The frequency of AF monitoring is in line with current clinical practice. We did not assess atrial strain rate as this was beyond the remit of our primary hypothesis.

The LA strain values of controls presented in this paper differ from some of the wider literature. However, our healthy controls were older, obese, and matched to HCM cohort (for blood pressure) and thus different to controls from other studies. These factors may explain potential differences in magnitude of strain. Another reason for differences CMR strain measurements across studies is the lack of standardisation in strain measurements across different analysis platforms. The increasing use of fully automated machine learning algorithms is expected to significantly improve standardisation and reduce interobserver variability, at which point CMR analysis of the LA strain could become a valuable clinically tool for risk prediction of AF. Finally, we did not undertake external validation of the thresholds for LA parameters and age, hence the selected thresholds require cautious interpretation when extrapolating to all HCM patients. Nevertheless, the present study provides novel insights into the additional value of LA deformation analysis on CMR for stratifying HCM patients at risk of new onset AF.

## Conclusion

We used CMR in HCM and demonstrate that LA strain components are reduced in patients who develop AF. Specifically, reservoir and booster LA strain augment risk prediction of new onset AF in HCM patients. Our work underscores the importance of age as a guide to arrhythmia surveillance and suggests that the routine assessment of LA strain in HCM patients could augment existing tools for AF and potentially stroke prediction in HCM.

## Supplementary Information

**Additional file 1: Table S1.** Reproducibility of strain parameters. **Table S2.** Diagnostic accuracy of predicting new onset AF. **Table S3.** Differences in age and rate of hypertension between those with and without sarcomeric variants. **Figure S1.** Flow chart showing inclusion of subjects. **Figure S2.** Survival curve for HCM patients.

## Data Availability

The datasets used and/or analysed during the current study are available from the corresponding author on reasonable request.

## Abbreviations

AF: Atrial fibrillation
AHA: American Heart Association
AUC: Area under curve
BMI: Body mass index
CMR: Cardiovascular magnetic resonance
CI: Confidence interval
ECG: Electrocardiogram
ESC: European Society of Cardiology
EF: Ejection fraction
HCM: Hypertrophic cardiomyopathy
HR: Hazard ratio(s)
ICC: Inherited Cardiac Conditions
LA: Left atrium/left atrial
LAEDV: Left atrial end-diastolic volume
LAEF: Left atrial ejection fraction
LAESV: Left atrial end-systolic volume
LASV: Left atrial stroke volume
LGE: Late gadolinium enhancement
LV: Left ventricle/left ventricular
LVEDV: Left ventricular end-diastolic volume
LVESV: Left ventricular end-systolic volume
LVOT: Left ventricular outflow tract
NPV: Negative predictive value
NYHA: New York Heart Association
PPV: Positive predictive value
ROC: Receiver Operator Characteristic
SCD: Sudden cardiac death
TTE: Transthoracic echocardiography
VUS: Variant of uncertain significance
VT: Ventricular tachycardia
