# Supplementary material for: Incremental value of left atrial booster and reservoir strain in predicting atrial fibrillation in patients with hypertrophic cardiomyopathy: a cardiovascular magnetic resonance study
Source: J Cardiovasc Magn Reson. 2021 Oct 11;23:109. doi: 10.1186/s12968-021-00793-6 (PMC8504076; doi:10.1186/s12968-021-00793-6)
Supplement: Supplementary file 1 — Additional file 1: Table S1. Reproducibility of strain parameters. Table S2. Diagnostic accuracy of predicting new onset AF. Table S3. Differences in age and rate of hypertension between those with and without sarcomeric variants. Figure S1. Flow chart showing inclusion of subjects. Figure S2. Survival curve for HCM patients. [file 12968_2021_793_MOESM1_ESM.docx]

# Additional

# Genetic screening

All patients underwent screening for 13 genes associated with hypertrophic cardiomyopathy (HCM) (*MYH7*, myosin heavy chain; *PLN*, phospholamban; *TNNI3*, cardiac troponin I; *TNNT2*, cardiac troponin T; *MYL2*, regulatory myosin light chain; *MYL3*, essential myosin light chain; *TPM1*, alpha tropomyosin; *MYBPC3*, myosin binding protein C; *ACTC1*, cardiac actin; *CSRP3*, muscle LIM protein; *FHL1*, four and a half LIM domains 1; *PRKAG2*, AMPK γ2; *GLA*, alpha galactosidase) including a screen (blood test) for mitochondrial mutations if no sarcomeric mutations were identified.

# Genetic classifications present and used in genetic association analysis

Individuals were split into sarcomeric variant carriers and non sarcomeric variant carriers. For most genes (*ACTC1*, *MYH7*, *MYL2*, *MYL3*, *TNNT2*, *TNNI3* and *TPM1*), individuals were considered to be sarcomere variant carriers if they possessed a VUS-indeterminate, VUS-favours pathogenic, likely pathogenic or pathogenic variant. Those possessing a non-truncating *MYBPC3* variant that was considered VUS-indeterminate were considered to be non sarcomeric variant carriers a previously described [1].

# Cardiovascular magnetic resonance (CMR) acquisition protocol

All patients had CMR imaging, including cine and late gadolinium enhancement (LGE) assessment at 1.5 or 3T. CMR field strength does not influence strain measurements [2]. Cine imaging was performed using standard methods, acquiring images encompassing the whole left atrium in the vertical long axis, left ventricular outflow tract (LVOT), and horizontal long axis planes and utilised single breath-hold balanced steady-state images as previously described [3]. LGE imaging of the left ventricle (LV) was acquired 8-10 minutes after IV infusion of the gadolinium-based contrast agent.

Cine images were acquired using a balanced steady state free precession imaging with TR/TE = 3.2/1.4ms; field of view = 380x273 mm, matrix 192x138, in-plane resolution 1.9x1.9 mm, slice thickness/spacing 7/3 mm; temporal resolution is 39-60 ms. T1-weighted phase-sensitive inversion recovery sequence was used for LGE imaging with TR/TE = 2.9/3.36 ms, field of view = 380x285 mm, matrix =256x144, slice thickness/spacing = 7/3 mm.

# Left atrial strain analysis

LA strain was measured using feature tracking techniques as previously described [4, 5]. CVI42 software (Circle Cardiovascular Imaging, Inc, Calgary, Alberta, Canada) was used to perform LA analyses. The 2- and 4-chamber long-axis views were used to trace the LA endocardial borders at LV end diastole. An automated tracking algorithm, designed to track thin walled structures (such as the right ventricle), was then applied to automatically propagate the contours throughout the entire cardiac cycle. To optimise wall tracking throughout the cardiac cycle, manual adjustments of contours were carried out where necessary. Neither the LA appendage nor pulmonary veins were included in the contours. Segmental deformation values from cvi42 software were exported and further processed. Reservoir, conduit and booster strain magnitude were then computed.

# Reproducibility of strain parameters

The intra-observer variability analysis for this method resulted in coefficients of variation of 13.0%, 15.91%, and 15.07% for the three measures of strain (**Table S1**). This was similar in the interobserver variability. The interclass coefficient of variability was 0.919, 0.884 and 0.745 for the intra-observer variability, and again was similar for the inter-observer values. As reservoir strain is the strain that is contoured, and conduit and booster inferred using modelling by the tracking software, we expected it to have the best measures of variability.

# Comparing age and rates of hypertension in those with and without sarcomeric variants

To explain the counter-intuitive effect of sarcomeric variant status on the risk of developing new onset atrial fibrillation (AF) we analysed the relationship between age, hypertension, and genetic status. Those with sarcomeric variants were younger, and less likely to have hypertension (**Table S3**).

# Additional tables

# Table S1: Reproducibility of strain parameters

|  | Intra-observer (RS vs RS) | | | Inter-observer (GT vs mean of RS) | | |
| --- | --- | --- | --- | --- | --- | --- |
|  | Mean Difference ± SD | CV (%) | ICC (95% CI) | Mean Difference ± SD | CV (%) | ICC (95% CI) |
| Reservoir strain | 0.82 ± 2.77 | 13.03 | 0.92 (0.49-0.98) | 1.12 ± 2.86 | 13.80 | 0.87 (0.27-0.97) |
| Conduit strain | 0.04 ± 2.12 | 15.91 | 0.88 (0.60-0.97) | 0.57 ± 2.29 | 17.57 | 0.88 (0.58-0.97) |
| Booster strain | 0.43 ± 1.37 | 15.07 | 0.75 (0.30-0.93) | 0.54 ± 1.18 | 13.37 | 0.77 (0.20-0.94) |
| SD, standard deviation; CV, coefficient of variation; ICC, intraclass correlation coefficient | | | | | | |

# Table S2: Diagnostic accuracy of predicting new onset AF

|  | | Diagnostic accuracy of the threshold values at 3 years post scan | |
| --- | --- | --- | --- |
|  | The threshold value | C statistic | Sensitivity, Specificity, NPV, PPV |
| Age at scan | ≥ 55 | 0.75 | 85, 54, 98,14 |
| LA diameter, mm | ≥ 45 (ESC guidelines) | 0.60 | 23, 92, 94, 19 |
|  | ≥ 42 |  | 38, 85, 93, 18 |
| LAEDV index, mL/m^2^ | ≥ 50 | 0.69 | 56, 72, 95, 15 |
| LAESV index, mL/m^2^ | ≥ 27 | 0.71 | 55, 75, 95, 16 |
| LA reservoir strain, % | ≤ 18 | 0.78 | 84, 57, 97, 15 |
| LA conduit strain, % | ≤ 12 | 0.66 | 94, 28, 98, 10 |
| LA booster strain, % | ≤ 8 | 0.71 | 73, 58, 96, 13 |
| LA ejection fraction, % | ≥ 45 | 0.70 | 67, 65, 94,14 |
| C-statistic, concordance statistic (equal to the area under the curve produce in ROC analysis); ESC, European Society of Cardiology; NPV, negative predictive value; PPV, positive predictive value.  Unless stated otherwise, the threshold is a ROC calculated optimal threshold value as per the Youden index. | | | |

# Table S3: Differences in age and rate of hypertension between those with and without sarcomeric variants

| In all patients, n=238 | | | |
| --- | --- | --- | --- |
|  | Sarcomeric variant carriers | Sarcomeric variant negative patients | P value (t-test) |
| Mean age at scan, years | 45 | 57 | <0.001* (t-test) |
| Hypertension, n, % | 12, 15% | 87, 57% | <0.001* (chi-squared) |
| No hypertension, n, % | 66, 85% | 66, 43% |  |

# Figure S1 Flow chart showing inclusion of subjects. Graphical depiction of the patients who had pre-existing AF and thus were excluded from the study, and the patients that developed new onset AF during the study.

#
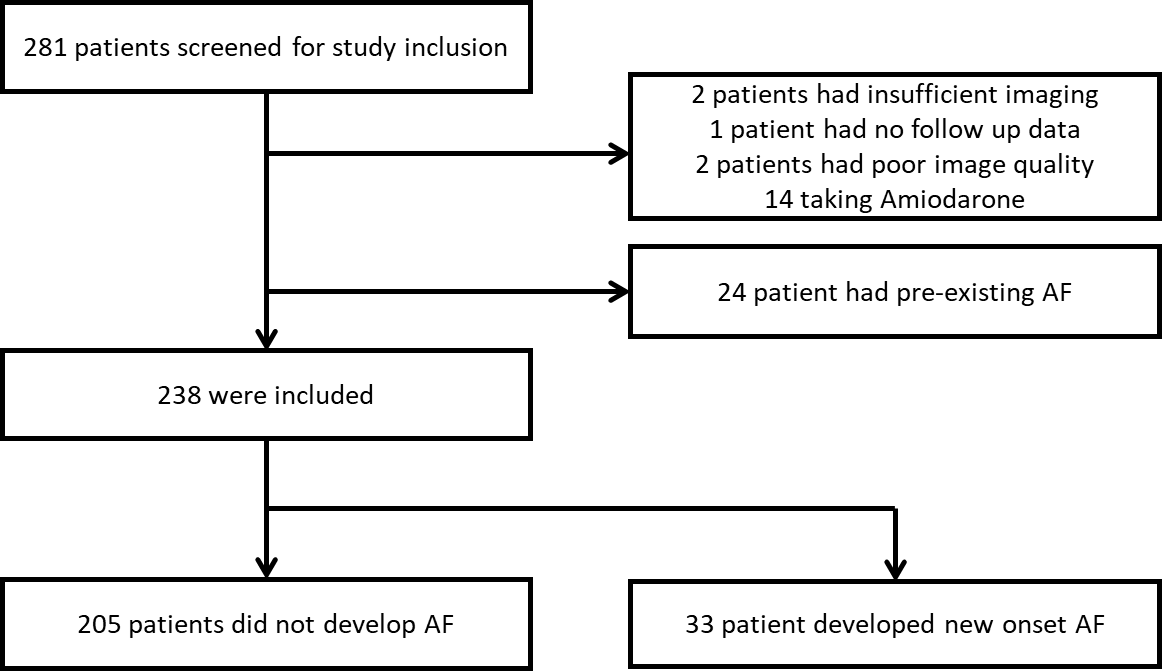


**
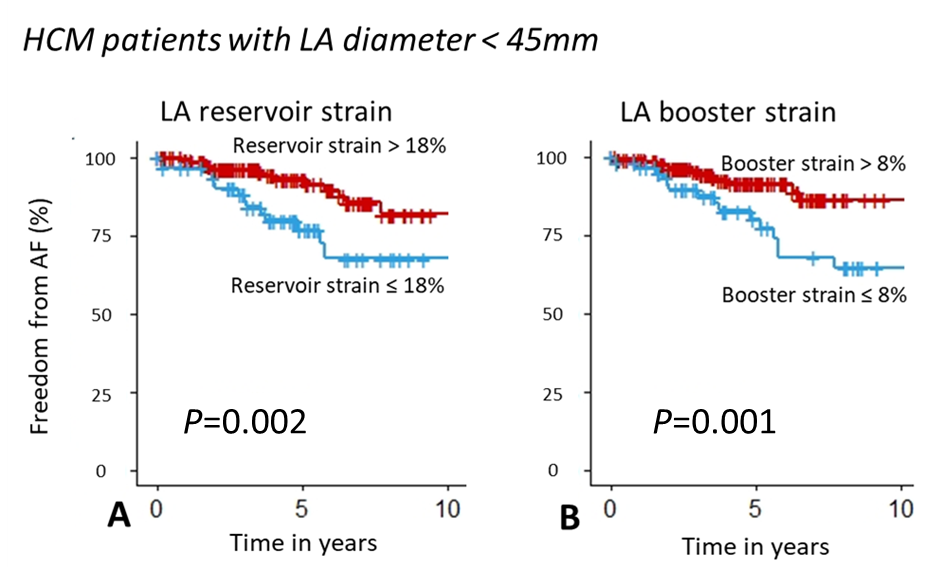
**

# Figure S2 Survival curve for HCM patients. Survival curves of all HCM patients (n=217) with LA diameter <45mm showing freedom from atrial fibrillation (AF) when stratified according to the variables that maintained significance in the Cox multiple regression model: A, Left atrial (LA) reservoir strain; B, LA booster strain

# References

[1] S. Neubauer *et al.*, "Distinct Subgroups in Hypertrophic Cardiomyopathy in the NHLBI HCM Registry," (in eng), *J Am Coll Cardiol,* vol. 74, no. 19, pp. 2333-2345, Nov 12 2019, doi: 10.1016/j.jacc.2019.08.1057.

[2] A. Schuster *et al.*, "The intra-observer reproducibility of cardiovascular magnetic resonance myocardial feature tracking strain assessment is independent of field strength," (in eng), *Eur J Radiol,* vol. 82, no. 2, pp. 296-301, Feb 2013, doi: 10.1016/j.ejrad.2012.11.012.

[3] S. Dass *et al.*, "Exacerbation of cardiac energetic impairment during exercise in hypertrophic cardiomyopathy: a potential mechanism for diastolic dysfunction," (in eng), *European heart journal,* vol. 36, no. 24, pp. 1547-54, Jun 21 2015, doi: 10.1093/eurheartj/ehv120.

[4] M. Imai *et al.*, "Multi-ethnic study of atherosclerosis: association between left atrial function using tissue tracking from cine MR imaging and myocardial fibrosis," (in eng), *Radiology,* vol. 273, no. 3, pp. 703-13, Dec 2014, doi: 10.1148/radiol.14131971.

[5] M. Evin *et al.*, "Left atrial aging: a cardiac magnetic resonance feature-tracking study," (in eng), *Am J Physiol Heart Circ Physiol,* vol. 310, no. 5, pp. H542-9, Mar 1 2016, doi: 10.1152/ajpheart.00504.2015.
